# Assessing Acute DWI Lesions in Clinically Diagnosed TIA: Insights from a Cohort Study in Cluj, Romania

**DOI:** 10.3390/tomography11040040

**Published:** 2025-03-27

**Authors:** Khaled Abu Arif, Ioan Stefan Florian, Alexandru Ioan Florian, Alina Vasilica Blesneag, Enola Maer, Răzvan Mircea Cherecheș

**Affiliations:** 1 Centrul Medical Transilvania SRL, 400486 Cluj-Napoca, Romaniaenolamaer@cmtransilvania.ro (E.M.); 2 Department of Clinical Neurosciences, Iuliu Hatieganu, University of Medicine and Pharmacy, 400012 Cluj-Napoca, Romania; 3 Department of Public Health, Babeș-Bolyai University, 400084 Cluj-Napoca, Romania

**Keywords:** transient ischemic attack, diffusion-weighted imaging, ischemic lesions, tissue-based diagnosis, stroke risk management

## Abstract

Background: The updated definition of a TIA emphasizes tissue characteristics rather than symptom duration, defining a TIA as a transient neurological episode without ischemic lesions in brain imaging, including in DWI. If imaging reveals a lesion, even in patients with transient symptoms, the event is reclassified as a minor ischemic stroke. Objective: This retrospective observational study aimed to determine the prevalence of ischemic lesions in DWI in patients with a TIA diagnosis. Results: Adults aged 18–90 years, diagnosed with a TIA by a neurologist and who underwent MRI-DWI at CMT hospital within the first week after symptom onset (May 2023–July 2024), were included. Ethical approval was obtained. Descriptive statistics summarized patient demographics, clinical features, Fazekas scale grades, and imaging findings. Conclusions: Among the 26 patients clinically diagnosed with TIAs, 7 (26.9%) exhibited ischemic lesions in DWI, reclassifying these cases as minor ischemic strokes under the updated definition. The prevalence of ischemic lesions was notably higher in patients with comorbidities such as hypertension and diabetes. These findings highlight the importance of early MRI-DWI to accurately distinguish TIAs from minor ischemic strokes. Routine urgent DWI within the first week of TIA symptoms enhances diagnosis and risk stratification and can guide targeted stroke prevention strategies, particularly when combined with the ABCD2 score.

## 1. Introduction

A transient ischemic attack (TIA) is a medical emergency characterized by a temporary episode of neurological dysfunction caused by localized ischemia in the brain, spinal cord, or retina that does not result in acute infarction or tissue damage. Most TIAs last less than an hour, often just a few minutes [1].

The definition of a TIA has evolved from being time-based, focusing on symptom resolution within 24 h, to emphasizing tissue involvement detected through advanced imaging techniques such as MRI-DWI.

Diffusion-weighted imaging (DWI) has become an essential tool for detecting ischemic changes in patients with transient ischemic attack (TIA) and minor stroke, offering greater sensitivity than computed tomography (CT), particularly in the early stages of ischemic injury. This advanced imaging modality is highly effective in identifying smaller lesions and lesions located in anatomically challenging areas such as the posterior circulation [2]. Studies indicate that DWI alone detects most ischemic changes, and its sensitivity is further enhanced when combined with perfusion-weighted imaging (PWI), reaching up to 97.5% in the diagnosis of ischemic stroke. Incorporating DWI into routine TIA evaluation provides significant clinical benefits, enabling earlier diagnosis, improved risk stratification, and targeted secondary prevention strategies. However, limitations such as delayed imaging or false negatives in posterior circulation strokes must be considered. Overall, DWI plays a pivotal role in modern stroke care, offering both high diagnostic precision and timely intervention opportunities [3].

The European Stroke Organisation (ESO) defines a transient ischemic attack (TIA) as transient neurological symptoms, likely due to focal cerebral or ocular ischemia, that last less than 24 h [4].

Patients suffering a TIA are clinically unstable and require prompt evaluation to reduce the risk of stroke [5,6]. Accurately identifying which patients are at the highest and lowest risk of stroke in the critical days and weeks following a TIA is essential for implementing effective secondary prevention measures.

One multicenter study examined the early stroke risk after transient ischemic attack (TIA) and evaluated the ABCD2 score’s predictive value, comparing tissue- and time-based TIA definitions. Among 4574 patients, tissue-positive events identified by DWI carried a higher 7-day stroke risk (7.1%) than tissue-negative events (0.4%), highlighting the importance of tissue-based criteria. The ABCD2 score was an effective predictor of stroke risk in both groups, with higher scores linked to greater risk. While CT imaging provides prognostic value, it is less sensitive than DWI in detecting tissue-positive cases [7]. These results emphasize the value of integrating tissue-based definitions and clinical scores for better stroke risk assessment and management. Incorporating MRI-DWI and neurovascular imaging, together with tools such as the ABCD2 score, enhances the prediction of ischemic stroke following a TIA [4].

Early and accurate imaging is critical in evaluating transient ischemic attacks (TIAs) and acute ischemic infarcts. Brain MRI, including DWI, is considered the most effective diagnostic tool and is best performed within 24 h of symptomatology. In many cases, NCCT is performed first; however, in centers with immediate MRI access, NCCT may be unnecessary for stable patients whose symptoms have fully resolved [8]. The likelihood of detecting ischemic lesions increases with the increase in symptom duration, with DWI detecting lesions in approximately 40% of patients with TIA symptoms [9].

MRI-DWI plays a pivotal role in the early evaluation of TIA, detecting acute ischemic lesions in approximately 34% of cases [10]. DWI-positive findings are associated with a higher risk of early stroke recurrence, enabling more precise risk stratification and informing targeted secondary prevention strategies. This imaging modality enhances diagnostic accuracy, distinguishing true ischemic events from mimics, and supports timely, evidence-based interventions to reduce recurrent stroke risk and improve outcomes.

MRI-DWI has further advanced the understanding and diagnosis of TIAs. Unlike the traditional time-based definition, MRI-DWI enables the detection of ischemic lesions in a substantial proportion of patients, often exceeding previously reported rates. These findings are frequently associated with a higher clinical severity, as reflected by the increased National Institutes of Health Stroke Scale (NIHSS) and ABCD2 scores, which are often linked to perfusion deficits and vascular occlusion. By providing a more sensitive method for detecting ischemic brain injury, MRI-DWI supports the transition toward a tissue-based definition of TIA, offering critical implications for risk stratification and the development of targeted secondary prevention strategies.

The usefulness of MRI in patients presenting with transient or minor neurological symptoms remains an area of ongoing investigation. Our objective was to assess the proportion of participants with these symptoms who exhibited MRI evidence of acute ischemia across varying clinical probabilities of TIA or minor stroke. While previous studies have explored TIA diagnosis and acute DWI lesions, this study focuses on a cohort in Cluj, Romania, providing insights into the application of tissue-based definitions of TIA in outpatient settings.

In Romania, access to MRI machines is limited [11], with approximately 336 machines available nationwide, of which only 56 are in the public sector. This restricted availability means that advanced imaging modalities such as MRI-DWI are primarily accessible in larger cities, creating disparities in diagnostic capabilities for TIA patients across the country. Moreover, there are no strict national guidelines for imaging in TIA cases, which further impacts the consistency and frequency of imaging investigations for these patients. These factors significantly influence the generalizability of the study’s findings, as the use of DWI for TIA diagnosis remains uncommon in routine clinical practice in many parts of Romania. Consequently, this study highlights the importance of addressing these disparities to improve diagnostic precision and patient outcomes in resource-limited settings.

## 2. Materials and Methods

### 2.1. Study Design

This was a retrospective observational study aimed at assessing the prevalence of ischemic lesions in DWI in adult patients in Cluj, Romania, with a clinical diagnosis of TIA without infarction on a CT scan. The study was designed to provide insights into the correlation between clinical diagnoses of TIA and the imaging evidence of ischemic lesions on DWI, further contributing to the understanding of the diagnostic utility of MRI in these cases.

### 2.2. Study Population and Sample

This study targeted adults aged 18–90 years residing in Cluj County, Romania, who underwent MRI at CMT. The inclusion criteria were as follows: a clinical diagnosis of TIA made by a neurologist and a DWI MRI scan within the first week following the TIA episode to ensure the reliability of the imaging findings. All patients with a clinical diagnosis of TIA who underwent MRI at CMT during the study period were assessed for eligibility.

As part of the inclusion process, every patient was required to complete a detailed questionnaire after being enrolled in the study. The questionnaire collected relevant medical history, including comorbidities, prior cerebrovascular events, medication use, and other pertinent clinical data. This structured approach ensured consistency in the collected data and allowed for a comprehensive analysis of potential influencing factors.

To maintain data integrity and ensure the completeness and accuracy of all records, data cleaning procedures were implemented. Missing or incomplete data points were excluded from the analysis to minimize bias and uphold the reliability of the study findings. Potential confounders, such as the timing of MRI, pre-existing medical conditions, and ongoing treatments, were identified; however, adjustments could not be performed due to the exploratory nature of the study and the relatively small sample size. The final sample size was determined by the number of patients meeting the inclusion criteria during the specified study period.

As this was a retrospective study, no follow-up visits or additional counseling were conducted for participants. Furthermore, reclassification of cases from TIA to stroke did not involve direct patient interactions, and no interventions were made based on these findings. Data were fully anonymized, ensuring ethical handling of participant information while maintaining clinical relevance for interpretation and reporting.

### 2.3. Data Collection

The data collection period spanned from May 2023 to June 2024, with participants providing demographic information and medical history through a structured questionnaire. These questionnaires captured details on patient age, sex, comorbidities (e.g., hypertension, diabetes, thrombophilia, and atrial fibrillation), medication history, and prior cerebrovascular events. This standardized approach facilitated the collection of robust data, allowing for meaningful subgroup analyses where appropriate.

In addition to questionnaire data, DWI MRI results were reviewed to confirm the presence or absence of ischemic lesions.

The MRI scanners utilized in our study were as follows:

- Philips Ingenia 3.0 T Omega HP (Philips Medical Systems, Amsterdam, The Netherlands): a 3.0 Tesla system featuring a 70 cm bore, Omega HP gradients (maximum amplitude 45 mT/m; slew rate 200 T/m/s), parallel RF transmission technology, dual 18 kW RF amplifiers, and a maximum FOV of 55 cm.
- GE Optima MR360 Advance 1.5 T (GE Healthcare, Chicago, IL, USA): a 1.5 Tesla MRI system with a 60 cm bore, gradients with an amplitude of 33 mT/m and a 120 mT/m/s slew rate, OpTix RF digital signal technology, and advanced imaging applications including 3D Cube and IDEAL Fat/Water Imaging.

For the 3 T scanner, DWI was performed using an axial single-shot echo-planar imaging sequence with the following acquisition parameters: field of view, 240 × 240 mm; in-plane voxel size, 0.65 × 0.65 mm; matrix size, 368 × 368. The slice thickness was set to 4 mm with an inter-slice gap of 1 mm, covering a total of 30 slices.

The repetition time and echo time were 4472 ms and 126 ms, respectively. The number of signal averages was set to 1. The total scan time for the sequence was 0.57 min (~34 s).

For the 1.5 T scanner, 1.5 T diffusion-weighted imaging (DWI) was performed using an axial acquisition with a field of view (FOV) of 250 mm. The voxel size was set to 0.195 × 0.195 × 0.4 mm, with an acquisition matrix of 128 × 128. A slice thickness of 4 mm with an inter-slice gap of 0.4 mm was used, covering a total of 34 slices. The number of signal averages (NSA) was set to 2 to enhance the signal-to-noise ratio (SNR). The echo time (TE) was set to the minimum within a range of 91–165 ms, while the repetition time (TR) was set to automatic within a range of 4000–12,000 ms. The total acquisition time for the sequence was 1 min and 20 s.

These MRI scans were systematically analyzed by two experienced radiologists. The lesion size, location, and their correlation with clinical presentation were documented to provide a comprehensive overview of ischemic patterns in the study population.

### 2.4. Statistical Analysis

Descriptive statistics, including means, standard deviations, and frequencies, were utilized to summarize the demographic and clinical data. These analyses were essential for characterizing the sample and estimating the prevalence of ischemic lesions identified among participants upon DWI. Where the data permitted, subgroup analyses were conducted to explore additional factors associated with the presence of ischemic lesions, providing deeper insights into the relationships between clinical variables. Due to the small sample size and the need for accurate *p*-value calculations, Fisher’s Exact Test was employed to evaluate the association between categorical variables, such as Fazekas grades and the presence of ischemic lesions.

Statistical analyses were performed using RStudio (version 4.4.2), a versatile and widely used software environment for statistical computing and graphics. RStudio allowed for detailed computations, ensuring accuracy and reproducibility in data processing and visualization. Microsoft Office tools were used for the organization and presentation of tables and figures, ensuring clarity and accessibility of results. Using these tools, the findings were presented in a structured and reproducible manner, facilitating their interpretation and application.

### 2.5. Ethical Considerations

Written informed consent was obtained from all participants, ensuring that they were fully informed about the study objectives, procedures, and their rights as participants. Confidentiality was safeguarded by anonymizing participant data and storing it securely on password-protected computers accessible only to authorized personnel. The study strictly adhered to ethical guidelines, and approval for the study protocol was obtained from the relevant ethics committee, ensuring compliance with all regulatory and ethical standards.

### 2.6. Use of AI in Manuscript Preparation

We utilized the AI language model ChatGPT-3.5 and ChatGPT-4 to assist in refining the manuscript’s language and improving the clarity of the content.

## 3. Results

### 3.1. Prevalence of Ischemic Lesions

**Figure 1:**
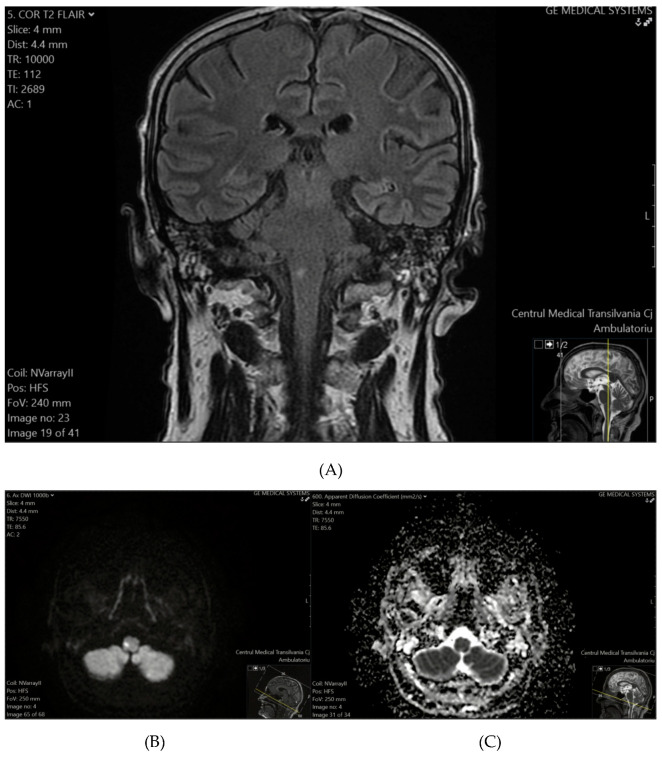
(**A**) (top) T2-FLAIR sequence reveals a hyperintense focus on the right side of the medulla oblongata. (**B**) (bottom-left) DWI confirms an ischemic event, demonstrating diffusion restriction with a hyperintense signal. (**C**) (bottom-right) The corresponding ADC map shows a hypointense signal, further supporting the presence of acute ischemia.

**Figure 2:**
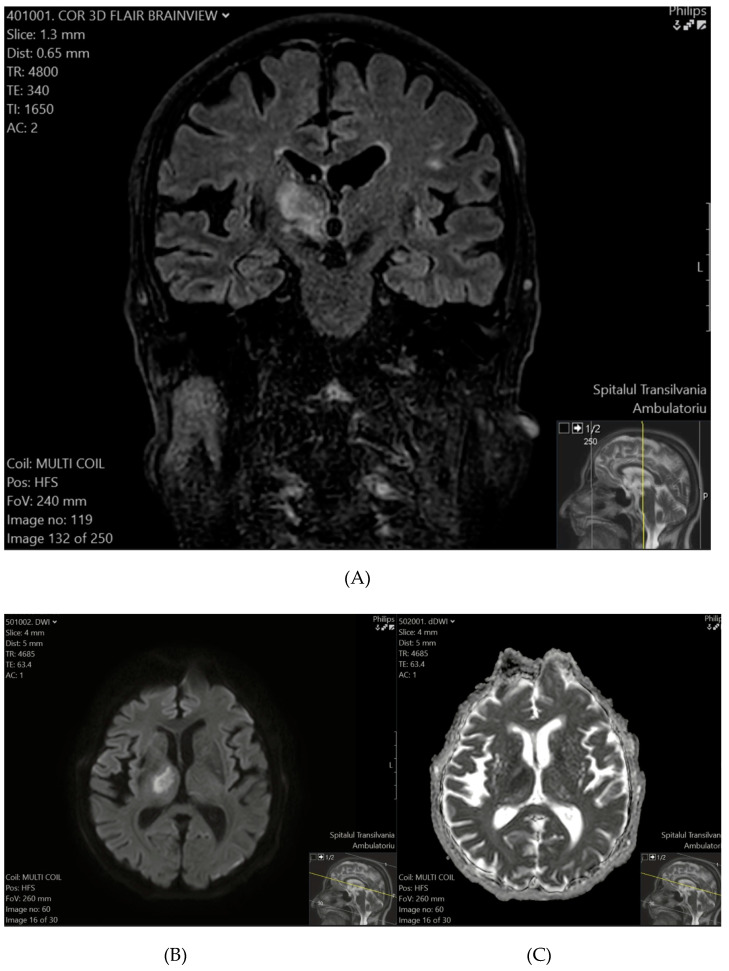
(**A**) (top) T2-FLAIR shows a hyperintense lesion in the right thalamus and the posterior limb of the internal capsule. (**B**) (bottom-left), (**C**) (bottom-right) DWI (**B**) and ADC (**C**) sequences confirm diffusion restriction, consistent with a recent ischemic event.

Ischemic lesions were identified in 26.92% of patients (95% CI: 11.6–47.8%), with a higher prevalence observed among older individuals, males, and those with a history of hypertension. These findings suggest that nearly one in four patients in the study cohort showed evidence of ischemic lesions, highlighting the importance of incorporating advanced imaging techniques to detect such lesions and understand their clinical significance in targeted patient subgroups. Representative MRI images from two patients illustrate characteristic ischemic lesions identified in our study cohort (Figure 1 and Figure 2). In Patient 1, a hyperintense lesion was observed in the right medulla oblongata on T2-FLAIR (Figure 1A), with corresponding diffusion restriction on DWI (Figure 1B) and a hypointense ADC signal (Figure 1C), confirming acute ischemia.

Patient 2 exhibited a hyperintense lesion in the right thalamus and the posterior limb of the internal capsule on T2-FLAIR (Figure 2A), with restricted diffusion on DWI and ADC sequences (Figure 2B,C), consistent with a recent ischemic event.

### 3.2. Sex Distribution

The study cohort consisted of 26 patients, with 46.15% (*n* = 12) being female and 53.85% (*n* = 14) male, indicating a slightly higher proportion of male participants. This modest male predominance aligns with trends often observed in similar populations.

### 3.3. Age Distribution

**Figure 3:**
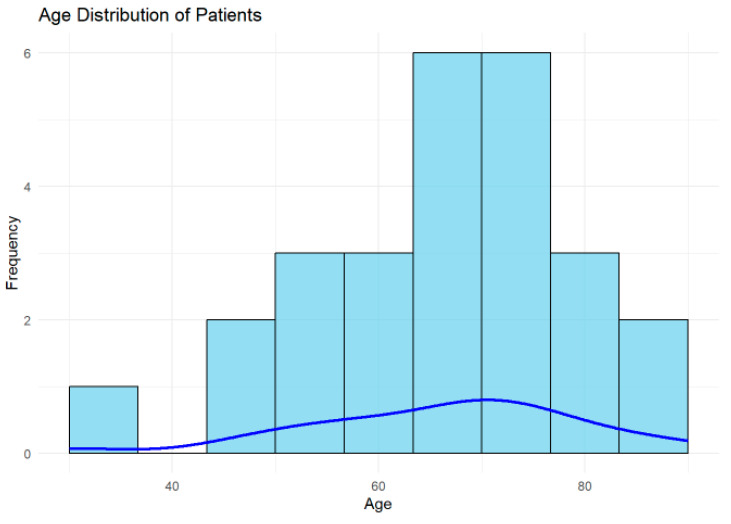
Histogram displaying the age distribution of patients, with a blue density curve representing the smoothed estimate. Most patients were aged 60–70 years, indicating that the cohort predominantly consisted of older adults.

The mean age of the cohort was 66.19 years (SD = 13.49 years), with a median age of 68.5 years, as shown in Figure 3. The interquartile range (IQR) was 16 years, reflecting a moderate spread in the ages of the studied population. These findings indicate that the cohort predominantly included older adults.

A histogram is presented to illustrate the distribution of patient ages in the cohort, with the blue density curve providing a smoothed estimate of the age distribution. The highest frequency of patients is between 60 and 70 years, indicating this is the most common age group in the cohort. The distribution indicates that most patients in the cohort are older adults, which is consistent with the higher prevalence of ischemic lesions and conditions related to TIA in older populations.

Younger patients (30–40 years) may represent outliers or individuals with unique risk factors or atypical presentations, warranting further investigation.

The age and risk factor distributions underscore the importance of targeting hypertension and vascular risk factors in this population.

### 3.4. Distribution of Risk Factors

**Figure 4:**
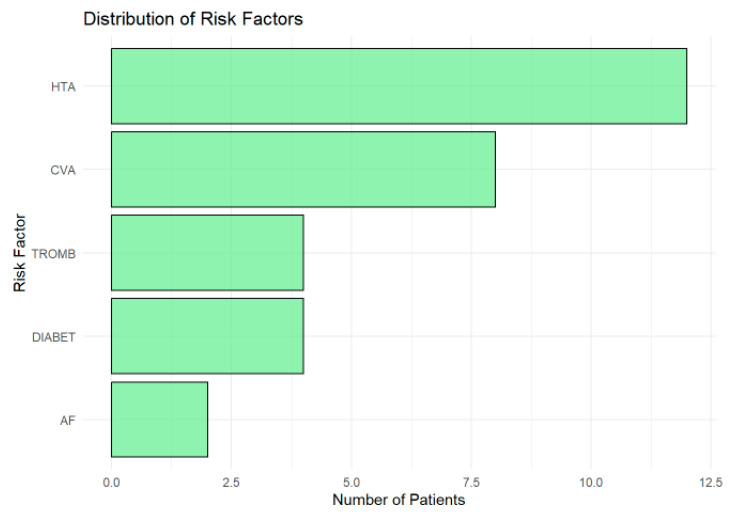
Horizontal bar chart showing the prevalence of risk factors in the cohort. Hypertension (HTA) was the most common, followed by a history of cerebrovascular accidents (CVAs). Thrombophilia (TROMB) and diabetes (DIABET) were moderately prevalent, while atrial fibrillation (AF) was the least frequent.

The distribution of risk factors (Figure 4) in the cohort highlights hypertension (HTA) as the most prevalent, affecting approximately 12 patients, underscoring its role as a major modifiable risk factor for ischemic lesions and cerebrovascular diseases. A history of cerebrovascular accidents (CVAs) was the second most common risk factor, present in around nine patients, reflecting the vulnerability of the cohort to recurrent ischemic events and the need for robust secondary prevention strategies.

Thrombophilia (TROMB) and diabetes (DIABET) were moderately prevalent, affecting about 4–5 patients each, highlighting the importance of addressing coagulation and metabolic risks in this population. Atrial fibrillation (AF), although less common (observed in fewer than three patients), remains clinically significant due to its strong association with cardioembolic strokes. Its low frequency in this cohort may reflect underdiagnosis or cohort-specific characteristics.

### 3.5. Frequency of Clinical Diagnoses

**Figure 5:**
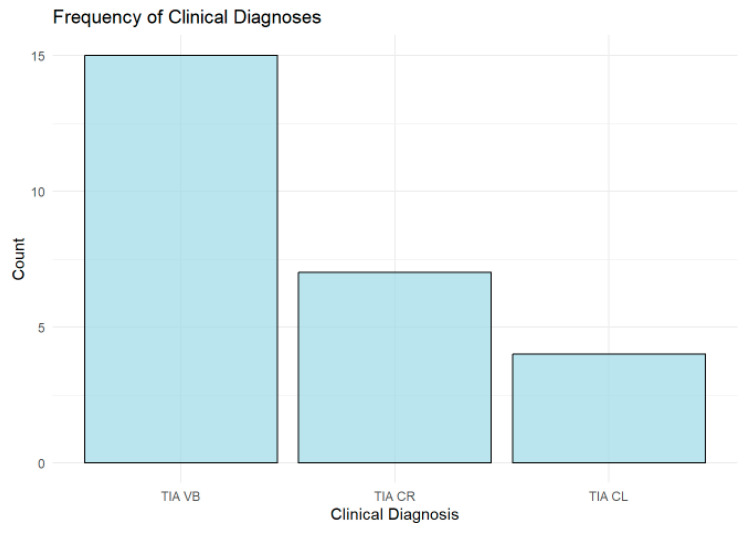
Bar chart showing the distribution of transient ischemic attack (TIA) types by vascular territory. Vertebrobasilar (TIA VB) was the most common, followed by right carotid (TIA CR), while left carotid (TIA CL) was the least frequent.

The analysis of clinical diagnoses shows that the vertebrobasilar (TIA VB) type is the most common, accounting for 57.69% (*n* = 15) of the cohort, followed by right carotid (TIA CR) cases at 26.92% (*n* = 7), while left carotid (TIA CL) cases are the least frequent at 15.38% (*n* = 4), as shown in Figure 5.

### 3.6. Distribution of Symptoms

**Figure 6:**
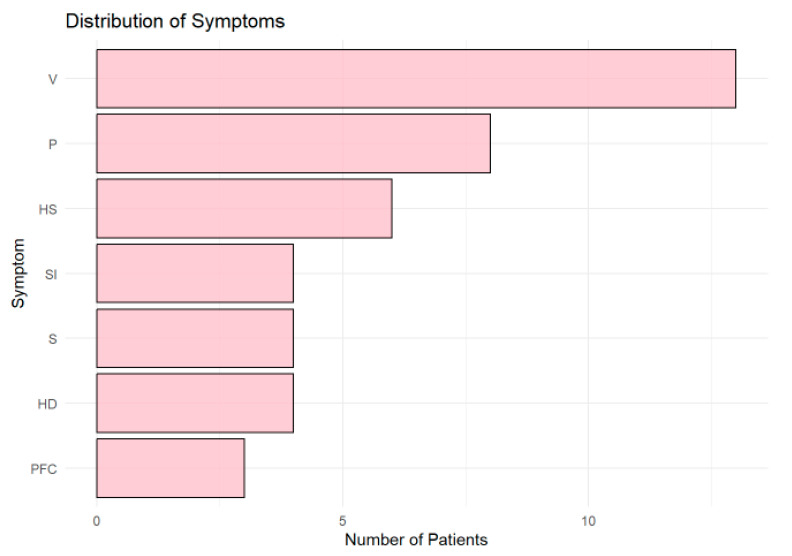
Horizontal bar chart showing the distribution of symptoms across patients. Vertigo (V) was the most common symptom, followed by paresthesia (P) and left-sided hemiparesis (HS). Other symptoms, including speech (SI) and sight impairment (S), were less frequent, while central facial palsy (PFC) was the least common.

Vertigo (V) was the most frequently reported symptom (Figure 6), observed in more than 10 patients, followed by paresthesia (P) and left-sided hemiparesis (HS). Other symptoms, including speech impairment (SI), sight impairment (S), and right-sided hemiparesis (HD), were moderately represented. Central facial palsy (PFC) was the least common symptom.

### 3.7. Fazekas Scale Distribution and Association with Ischemic Lesions

**Figure 7:**
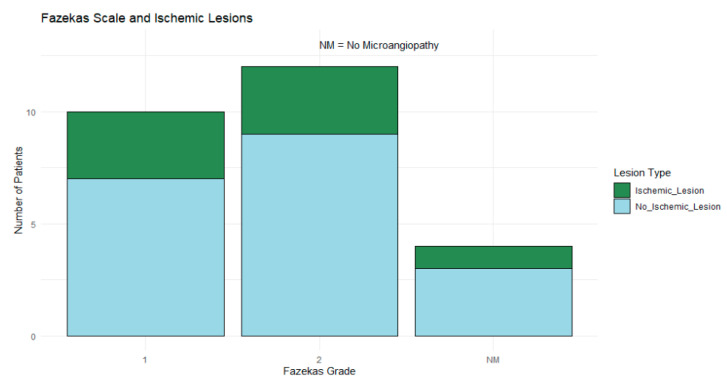
Stacked bar chart showing ischemic (blue) and nonischemic (green) lesions across Fazekas grades (1, 2, and NM). Fazekas grade 2 was most common, but no clear pattern emerged in lesion distribution.

Fazekas grade 2 was the most common, observed in 46.15% (*n* = 12) of patients, followed by grade 1 (38.46%, *n* = 10), while 15.38% (*n* = 4) of patients showed no microangiopathy. Ischemic lesions were observed across all Fazekas grades, though no definitive pattern emerged in the distribution, as shown in Figure 7.

Fisher’s Exact Test was applied to a contingency table to compare patients with ischemic lesions to those without lesions across different Fazekas grades. This analysis aimed to detect any statistically significant associations between microangiopathy severity and ischemic lesions given the categorical nature of these variables and the limited sample size; however, it did not yield statistically significant findings (*p* > 0.05), suggesting no clear association between the Fazekas grades and ischemic lesions in this cohort.

## 4. Discussion

The rate of acute DWI lesions observed in our study (27% of TIAs) is consistent with previously reported ranges [12,13,14,15]. Evidence from prior studies reaffirms the critical role of DWI in detecting ischemic lesions in TIA cases, with positivity rates ranging from approximately 17% to 67% [16]. By providing a more sensitive method for detecting ischemic brain injury, DWI supports the transition toward a tissue-based definition of TIA, with important implications for risk stratification and the development of targeted secondary prevention strategies. However, DWI’s reduced sensitivity in specific scenarios, such as for small lesions or those located in the posterior circulation, highlights the need for cautious interpretation, as false negatives may occur. Advanced imaging techniques, such as perfusion imaging or the combined analysis of DWI and ADC maps, could further improve the diagnostic accuracy, particularly for atypical presentations [16]. Additionally, DWI findings provide valuable insights into stroke etiology; for instance, patterns of multiple lesions in different vascular territories often suggest cardioembolic sources [16]. These insights not only inform secondary prevention strategies but also underscore the importance of future research to assess the prognostic value of lesion characteristics in refining diagnostic and therapeutic approaches for TIAs and minor strokes.

While our findings regarding the detection of acute DWI lesions in patients with a clinical diagnosis of TIA are consistent with previous reports, our study offers valuable confirmatory evidence from a unique geographical and resource-limited setting. Our data underscore the practical challenges and clinical implications of applying tissue-based diagnostic criteria in Romania—where the availability of MRI scanners is limited and national imaging guidelines for TIA remain underdeveloped. Additionally, the implementation of routine advanced imaging protocols such as MRI-DWI or alternative modalities such as CT perfusion depends not only on equipment availability but also on the presence of adequately trained healthcare professionals, including radiologists and MRI technicians. Attracting and retaining such specialized personnel in smaller, remote hospitals remain challenging due to limited professional incentives and infrastructure support. Future healthcare policies should therefore address incentives to attract well-trained professionals to remote or smaller hospitals, which could substantially improve stroke diagnosis and prevention and overall patient outcomes in these settings. As mentioned in the literature, transitioning to a tissue-based definition of TIA using DWI provides valuable prognostic insights. All acute DWI lesions were included in this analysis, regardless of their alignment with clinical symptoms, acknowledging that symptoms do not always correspond directly to tissue localization due to factors that remain unclear. Very little has been reported in the literature on the occurrence of symptoms that do not correspond to the vascular territory of ischemia, especially over the past decade, raising important questions about the underlying mechanisms and diagnostic challenges in such cases, particularly among patients with atypical or ambiguous presentations. Including all DWI lesions ensures a more comprehensive understanding and reduces the risk of overlooking atypical findings. Future studies could focus on distinguishing between “clinically relevant” and “incidental” lesions to refine the reclassification of TIA and minor stroke, ultimately enhancing the diagnostic accuracy and guiding patient management.

The detection of ischemic lesions in MRI-DWI justifies more aggressive secondary prevention to reduce early stroke recurrence, particularly dual antiplatelet therapy (DAPT) (aspirin + clopidogrel) for 21 days, as supported by the CHANCE and POINT trials. Beyond antiplatelets, intensified management of hypertension, high-intensity statins, and anticoagulation in atrial fibrillation are crucial. Routine vascular imaging should guide decisions on carotid endarterectomy or stenting in cases of significant stenosis. These findings highlight the importance of early MRI-DWI to optimize risk stratification and refine stroke prevention strategies.

The existing literature indicates that TIA patients with acute ischemic changes on DWI face an increased 90-day stroke risk, possibly due to a heightened vulnerability of brain tissue to infarction [17]. In our cohort, individuals with DWI-positive lesions were more frequently males aged 60–80 years and demonstrated a trend toward a higher prevalence of hypertension (HTA).

The strengths of this study include that TIA diagnoses were confirmed by a neurologist and the imaging results were thoroughly radiologically evaluated. However, there are limitations that warrant consideration. Previous studies have shown that TIA diagnoses based solely on clinical notes often result in moderate agreement among stroke specialists, highlighting the subjectivity of diagnostic thresholds. The type of scale also influences ratings, particularly for “unlikely TIA” cases, where substantial disagreement may impact patient management. These findings emphasize the need for standardized criteria to improve consistency [18].

We acknowledge that our study’s retrospective design, small sample size (*n* = 26), absence of a control group, and lack of long-term follow-up data limit the statistical robustness and generalizability of the findings. These methodological constraints are inherent to exploratory studies in resource-constrained settings and reflect the real-world challenges of accessing advanced imaging in parts of Romania. Nonetheless, our approach provides a preliminary but important dataset that can serve as a catalyst for larger, prospective studies employing multivariate analyses (e.g., logistic regression or survival analysis) to better adjust for potential confounders.

The relatively small sample size and the predominantly Caucasian population limit the generalizability of the findings to more diverse populations [19]. We further recognize that potential confounders—such as variability in the timing of MRI-DWI, pre-existing cerebrovascular diseases, and the severity of microangiopathic changes—could have influenced lesion detection. Specifically, delays in MRI-DWI (up to 7 days after symptom onset) may have allowed the reversal of transient ischemic lesions or the emergence of new clinically silent lesions, potentially affecting lesion prevalence estimates. This limitation is consistent with other studies, where delays in imaging have similarly affected the ability to detect acute ischemic lesions. Moreover, pre-existing cerebrovascular disease or pronounced microangiopathic changes (reflected by Fazekas grades) could confound lesion detection by complicating image interpretation or masking acute ischemic changes. Due to our limited sample size and retrospective approach, we were unable to perform multivariate analyses adjusting for these confounders. Additionally, ABCD2 scores were not available for all participants, limiting our ability to directly correlate clinical risk stratification with imaging findings. Future prospective studies should incorporate standardized imaging protocols, systematic recording of ABCD2 scores, detailed documentation of cerebrovascular disease history, microangiopathic severity, and standardized imaging timing to clarify these relationships and enhance their clinical relevance [20]. Despite these limitations, our findings remain clinically relevant, underscoring the potential importance of early MRI-DWI to identify patients who may benefit from intensified secondary stroke prevention strategies.

All patients underwent DWI using 1.5 T or 3 T MRI systems with a slice thickness of 4 mm. While this approach can reliably detect most ischemic lesions, the use of standard-resolution DWI across all cases may have reduced sensitivity for identifying smaller or more subtle lesions, particularly in regions such as the brainstem or posterior circulation where lesion size and anatomical complexity pose additional challenges. In our study, we were unable to directly calculate the sensitivity and specificity of DWI-MRI due to the retrospective design and the lack of a definitive gold-standard reference. However, prior research has consistently demonstrated the high diagnostic accuracy of DWI-MRI for detecting acute ischemic brain injury, with reported sensitivity ranging from 88% to 100% and reported specificity lying between 95% and 100% [3,21]. High-resolution DWI sequences, which offer an enhanced spatial resolution, could have better detected these subtle ischemic changes. This limitation should be considered when interpreting the findings, as some smaller lesions may have been missed, potentially leading to an underestimation of the true prevalence of ischemic events. Future studies employing advanced imaging protocols, such as thin-slice or high-resolution DWI, may help address these limitations and provide a more comprehensive evaluation of ischemic lesions in TIA and minor stroke populations.

In our study group, there was no significant difference in DWI-based lesion detection relative to the time after symptom onset, suggesting that lesion visibility remained consistent within the observed time window. We cannot be certain that these findings are fully applicable to hyperacute settings such as the emergency department, as our study primarily focused on an outpatient cohort. The time window of up to 7 days after symptom onset may have influenced the detection of DWI-positive lesions. Immediate imaging in the emergency department could identify more acute ischemic lesions, while delayed imaging might lead to some findings being missed. The implementation of rapid MRI protocols for acute ischemic stroke evaluation was associated with an 18.7% reduction in direct costs and a 17% decrease in hospital length of stay compared to CT perfusion, without impacting emergency department length of stay [22]. These findings suggest the potential cost-saving and efficiency benefits of rapid MRI, though further studies are needed to validate these preliminary results.

One study found that the likelihood of false-negative results on diffusion-weighted imaging (DWI) decreases as the time from transient ischemic attack (TIA) onset increases, with no false negatives observed beyond 3 h; thus, performing DWI within 2 h of symptom onset carries a higher risk of false-negative findings [14]. Conversely, delayed imaging could also detect new, clinically silent lesions that develop after the initial event, potentially overestimating the prevalence of lesions detected by DWI. These factors highlight the need for further studies in hyperacute settings to better understand the timing of optimal imaging for TIA patients.

In our study, we did not specifically account for potential false-negative DWI results, particularly in posterior circulation TIAs. A recent systematic review and meta-analysis highlighted that approximately 19% of posterior circulation ischemic strokes present with negative DWI findings compared to only 7% in anterior circulation strokes, with the former having a significantly higher risk of being DWI-negative (OR, 2.47; 95% CI: 1.30–4.72) [23]. Given this, it is possible that some of our patients classified as DWI-negative may have indeed experienced small, undetected infarcts. Future research should integrate more sensitive imaging strategies, including high-resolution MRI or repeat imaging, to accurately identify lesions.

The cohort consisted of participants presenting with transient or minor neurological symptoms in both emergency and outpatient settings. Clinicians at various levels of training provided a differential diagnosis, with a TIA or a stroke considered the most likely diagnosis prior to conducting a 1.5 or 3 T brain MRI within 7 days of symptom onset. MRI evidence of acute ischemia was determined based on two independent readings of the MRI scans.

The predominance of vertebrobasilar TIAs (57.69%) aligns with the observation that vertigo was the most frequently reported symptom in this cohort. This association highlights the involvement of the posterior circulation, as the vertebrobasilar territory supplies critical structures such as the brainstem and vestibular system. Ischemia in this region commonly manifests as vertigo due to transient dysfunction of the vestibular nuclei or their pathways [24].

Distribution factor findings emphasize the need for a multifaceted approach to risk management, with a primary focus on hypertension control and secondary prevention in patients with prior cerebrovascular events. Addressing metabolic, coagulation, and cardiac rhythm disorders remains critical for reducing the overall burden of ischemic lesions.

Identifying DWI-positive lesions in TIA cases underscores the need for more aggressive secondary prevention such as early initiation or intensification of antithrombotic therapy. These findings support routine DWI within 72 h to 1 week of a suspected TIA to improve risk stratification and guide management.

It is well known that a subset of TIA patients exhibit DWI-positive lesions, prompting reclassification as minor strokes. Our study reinforces this concept within a local clinical context, where diagnostic practices may vary significantly from those in centers with better imaging resources. By confirming the presence of ischemic lesions in approximately 27% of TIA cases, our findings emphasize the critical need for early MRI-DWI in routine practice. This reinforces existing clinical guidelines while also advocating for the development of national protocols to improve diagnostic accuracy and stroke prevention in settings with limited MRI access.

## 5. Conclusions

This study highlights the vital role of MRI-DWI in improving the diagnostic accuracy of transient ischemic attacks (TIAs), with 27% of cases reclassified as minor strokes due to the detection of ischemic lesions. Tissue-based definitions of TIA provide a more precise framework for risk stratification and facilitate the implementation of targeted secondary prevention strategies to reduce the risk of stroke recurrence. However, the limited availability of MRI machines, particularly in smaller or rural areas of Romania, and the lack of standardized imaging protocols for TIA remain significant challenges. These findings emphasize the need for improved access to advanced imaging and for the development of national guidelines to ensure consistent diagnostic practices.

Looking ahead, MRI-DWI should ideally be performed within the first 48–72 h of symptom onset to maximize sensitivity and diagnostic accuracy. Additionally, improved training for stroke neurologists is essential to enhance the interpretation of imaging findings and ensure appropriate management of TIA patients. Future research should validate these findings in larger, more diverse cohorts and explore the impact of timely imaging and specialized care on patient outcomes. By addressing these gaps, we can improve the precision of TIA management and reduce the global burden of stroke.

In conclusion, although our study reaffirms the established knowledge regarding the use of MRI-DWI in distinguishing TIA from minor ischemic stroke, it importantly contextualizes these findings within the framework of a resource-limited healthcare system. The challenges highlighted by our small sample size, lack of a control group, and short recruitment period provide a foundation for future studies. We think that our findings serve as a preliminary step toward refining imaging protocols and developing national guidelines that could improve stroke risk stratification and secondary prevention strategies in Romania.

## Data Availability

Given the retrospective nature of the study and potential ethical restrictions on patient data, the data presented in this study are not publicly available due to ethical and privacy considerations but may be available on reasonable request from the corresponding author, subject to Institutional Review Board approval.

## Abbreviations

The following abbreviations are used in this manuscript:

TIA	Transient Ischemic Attack
MRI	Magnetic Resonance Imaging
DWI	Diffusion-Weighted Imaging
NCCT	Non-contrast CT
MRI-DWI	Magnetic Resonance Imaging with Diffusion-Weighted Imaging
CT	Computed Tomography
